# Primer reporte de *Aedes* (*Stegomyia*) *albopictus* (Skuse) en la Orinoquia colombiana

**DOI:** 10.7705/biomedica.4344

**Published:** 2019-12-30

**Authors:** Malenna Camacho-Gómez, Liliana Patricia Zuleta

**Affiliations:** 1 Laboratorio de Entomología, Secretaría de Salud de Casanare, Yopal, Colombia Laboratorio de Entomología Secretaría de Salud de Casanare Yopal Colombia

**Keywords:** Aedes, vectores de enfermedades, mosquitos vectores, Aedes, disease vectors, mosquito vectors

## Abstract

**Introducción.:**

*Aedes albopictus* es vector de arbovirus, como *Flavivirus*, *Alphavirus*, *Bunyavirus*, *Phlebovirus*, *Orbivirus* y *Picornavirus.* Muchos son agentes etiológicos de enfermedades en humanos. Actualmente, *A. albopictus* se encuentra en expansión geográfica por su adaptación a diversos ambientes y tipos de criaderos. En Colombia, este mosquito fue reportado por primera vez en 1998 y, hasta el momento, se ha registrado en 10 departamentos.

**Objetivo.:**

Determinar la presencia de *A. albopictus* en Yopal, Casanare.

**Materiales y métodos.:**

En una búsqueda activa de larvas de *A. aegypti* en la zona industrial de Yopal, se observaron por primera vez mosquitos adultos de *A. albopictus*. Por lo anterior, se realizó la inspección en el intradomicilio y el peridomicilio de las viviendas en ocho localidades del municipio, en la cual se recolectaron larvas y pupas al inspeccionar hábitats larvarios, y hembras adultas, mediante capturas sobre atrayente humano protegido.

**Resultados.:**

Se identificaron 755 larvas de mosquitos, 71,5 % de *A. aegypti*, 24,8 % de *A.albopictus*, 3,2 % de *Culex quinquefasciatus* y 0,8 % de *C. coronator* y *C. nigripalpus*. Se capturaron 37 mosquitos adultos de *A. albopictus*. Los depósitos con mayor abundancia de este vector fueron las llantas.

**Conclusión.:**

Ante la presencia de *A. albopictus* se sugiere intensificar el sistema de vigilancia entomológica para detectar nuevas poblaciones dentro del departamento y en las áreas cercanas. Se debe poner atención a los criaderos artificiales de las zonas cercanas a los parqueaderos de vehículos de transporte de alimentos, insumos y maquinaria, procedentes de áreas con presencia del vector.

## Abstract (en)

**Introduction:**

*Aedes albopictus* is an arbovirus vector, such as *Flavivirus, Alphavirus*, *Bunyavirus*, *Phlebovirus*, *Orbivirus* and *Picornavirus*. Many are etiological agents of diseases in humans. Currently, *A. albopictus* is in geographical expansion due to its adaptation to different environments and breeding sites. In Colombia this mosquito was first reported in 1998 and currently it has been registered in 10 departments.

**Objective:**

To determine the presence of *A. albopictus* in Yopal, Casanare.

**Materials and methods:**

In a search of larvae of *A. aegypti* in the industrial zone of Yopal adult mosquitoes of *A. albopictus* were observed for the first time. Then, the inspection was carried out in the indoor and outdoor of houses in eight localities of the municipality, in which larvae and pupae were collected by inspection of breeding sites and adult females were captured by human landing catches.

**Results:**

Seven hundred and seventy-five larvae were identified, 71.5 % larvae of *A.aegypti*, 24.8 % of *A. albopictus*, 3.2 % of *Culex quinquefasciatus* and 0.8 % of *C. coronator* and *C. nigripalpus*. Thirty-seven adult mosquitoes of *A. albopictus* were collected. The most abundant breeding sites of this vector were tires.

**Conclusion:**

Given the presence of *A. albopictus* we suggest to intensify the entomological surveillance system to detect new populations within the department and nearby areas. Attention should be given to artificial breeding sites located in areas close to the parking of vehicles of transport of foods, supplies, and machinery from areas with the presence of the vector.

*Aedes* (*Stegomyia*) *albopictus* (Skuse) es vector, por lo menos, de 26 arbovirus. Entre estos están: *Alphavirus*, *Bunyavirus*, *Phlebovirus*, *Orbivirus*, *Picornavirus y Flavivirus* como los virus del dengue DENV-1, -2, -3 y -4 ^1^. Este mosquito tiene capacidad de transmitir de forma horizontal los virus del dengue, chikungunya y Zika ^1^^-^^3^. De igual forma, se ha registrado que puede transmitir verticalmente los virus del dengue y el Zika en Fortaleza, Camaçari y Belo Horizonte, en Brasil ^4^. También, se ha comprobado la capacidad de este mosquito de transmitir los parásitos de la malaria aviaria, *Plasmodium lophurae*^5^ y *P. gallinaceum*^3^.

*Aedes albopictus* es originario de los bosques del sudeste de Asia; sin embargo, se ha adaptado progresivamente a los ambientes antrópicos, con presencia en zonas rurales y urbanas en todos los continentes ^1^. Por lo anterior, es considerado como un vector capaz de transportar virus del ciclo silvestre al área urbana, como el Mayaro y el de la fiebre amarilla ^6^.

Su distribución en el planeta se debe, principalmente, a sus características fisiológicas, entre ellas, la diapausa en los huevos cuando están expuestos a temperaturas extremas. Las larvas pueden adaptarse a diferentes tipos de criaderos, desde naturales, como las axilas de plantas, hasta las artificiales, como las llantas. Las hembras se adaptan a un amplio espectro de fuentes alimenticias, como aves, mamíferos y humanos ^1^^,^^3^^,^^7^.

En 1985, esta especie fue reportada por primera vez en Houston, Texas, y en el hemisferio occidental ^3^. Posteriormente, en 1986, se registró en los estados de Espirito Santo, Minas Gerais, Rio de Janeiro y Sâo Paulo, en Brasil ^3^^,^^8^. Su introducción a Colombia fue registrada por primera vez en Leticia (Amazonas) en 1998, luego de su introducción en el estado de Tabatinga en Brasil en 1996 ^9^. Subsiguientemente, se reportó en Buenaventura (Valle) en 2001 ^10^, en Cali (Valle) en 2006 ^11^, en Medellín (Antioquia) en el 2011 ^12^, en Condoto e Istmina (Chocó), en los años 2011 y 2016, respectivamente, ^13^ y en La Tebaida (Quindío) ^14^. En este municipio, además, se llevó a cabo un estudio mediante el gen *COI* (*Cytochrome C Oxidase Subunit I*) en las poblaciones de *A. albopictus*, para detectar los orígenes geográficos de esta especie en Colombia. Los resultados revelaron una estrecha relación filogeográfica de estas poblaciones con las poblaciones de *A. albopictus* de Singapur y Los Ángeles, USA. Esto podría indicar que las poblaciones de este vector que circulan en el interior de Colombia llegaron inicialmente al puerto de Buenaventura, y que tienen un origen asiático ^14^.

Finalmente, el vector ha sido registrado en 10 departamentos del país, distribuidos en tres regiones geográficas (Pacífica, Andina y en el sur de la región Amazónica), de las seis que tiene Colombia ^13^.

En el departamento de Casanare, ubicado en la región de la Orinoquia, la vigilancia entomológica para detectar la aparición de *A. albopictus* se inició en el año 2004, según los informes del Laboratorio de Entomología de este departamento*.* La vigilancia se hizo mediante la instalación de cuatro larvitrampas en el perímetro cercano al aeropuerto del municipio de Yopal, las cuales eran revisadas semanalmente, recolectándose las larvas de culícidos para su identificación taxonómica. Posteriormente, fueron incluidos otros sitios, como el terminal de transporte y la plaza de mercado, considerados por la afluencia de personas, insumos y materiales procedentes de otras zonas del país.

En los 19 municipios de Casanare, también se hace la vigilancia entomológica de forma regular para determinar los índices entomológicos de *A. aegypti*. Esto se hace mediante la recolección e identificación de especímenes de culícidos provenientes de la inspección de los sitios de reposo de los mosquitos adultos y de los criaderos potenciales en los depósitos de agua, artificiales y naturales, más el muestreo con larvitrampas.

 El presente estudio se realizó con el objetivo de determinar la presencia de *A. albopictus* en Yopal, Casanare, e identificar sus posibles criaderos en el municipio.

## Materiales y métodos

### Área de estudio

**Figura 1.**
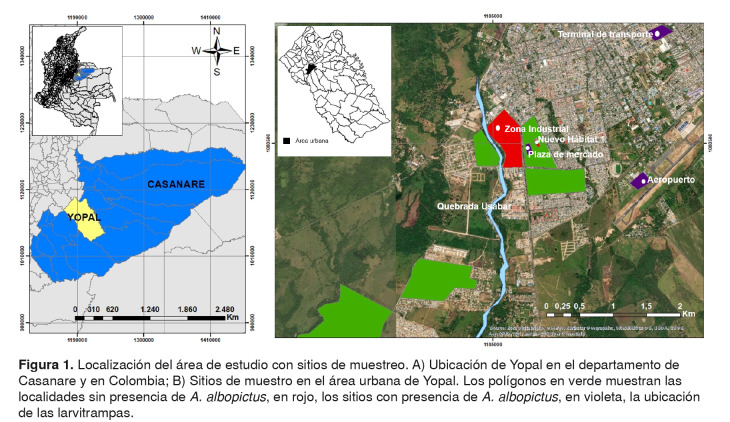
Localización del área de estudio con sitios de muestreo. A) Ubicación de Yopal en el departamento de Casanare y en Colombia; B) Sitios de muestro en el área urbana de Yopal. Los polígonos en verde muestran las localidades sin presencia de *A. albopictus*, en rojo, los sitios con presencia de *A. albopictus*, en violeta, la ubicación de las larvitrampas.

El municipio de Yopal, capital del departamento de Casanare, está ubicado en el piedemonte de la Cordillera Oriental de Colombia, en la latitud 5°19′50" N y la longitud 72°23'26" O, a 390 m.s.n.m.; pertenece al piso térmico cálido y su temperatura promedio anual es de 26,7 °C (15) (figura 1A). Según las proyecciones del Departamento Nacional de Estadística (DANE), en el 2017 el municipio contaba con una población de 146.204 habitantes y el 89 % habitaba en el área urbana ^16^, que se distribuye en cinco comunas y 114 barrios. Las principales actividades económicas de Yopal son la extracción petrolera, la ganadería y la agricultura, con cultivos de arroz, palma africana, plátano, maíz, café y yuca ^15^.

### Antecedentes

El 13 de diciembre de 2016, durante la búsqueda activa de estados inmaduros de *A. aegypti* en depósitos ubicados en un parqueadero de vehículos de carga de hidrocarburos y otros (5°19’14” N y 72°24’21” O), próximo a la plaza de mercado en la zona industrial del área urbana de Yopal, se detectó la presencia de mosquitos adultos de *A. albopictus* (figura 1B). Este lugar se caracterizaba por la presencia de llantas usadas, depósitos inservibles y la afluencia de vehículos de diferentes lugares del país.

Tras la detección de *A. albopictus*, se realizaron actividades de vigilancia entomológica en diciembre de 2016, para levantar los índices aédicos en 955 viviendas distribuidas en ocho localidades, la zona industrial y siete barrios ubicados en tres comunas cercanas al sitio donde se registró la especie por parte de los funcionarios de las Secretarías de Salud de Yopal y Casanare.

Se inspeccionaron todas las viviendas (N=720) de los barrios colindantes (Nuevo Hábitat 1, Siete de Agosto y El Laguito) con la zona industrial (figura 1B) y el 10 % (235) de las viviendas de los otros barrios (La Bendición, Llano Lindo, Villa Flor y Nueva Esperanza).

Sin embargo, aunque las localidades fueron categorizadas como urbanas, tres de los barrios (La Bendición, Llano Lindo y Villa Flor) se encuentran en la periferia y están en proceso de urbanización creciente.

### Determinación de los índices de infestación aédicos larvarios y de pupas

Se inspeccionaron los depósitos de agua, naturales y artificiales, de las viviendas, bodegas u otros sitios ubicados en cada localidad. La inspección se llevó a cabo en el interior de la vivienda o intradomicilio y en su peridomicilio, en un radio de 50 m, con el fin de registrar la presencia de especies de *Aedes*. Luego, se diligenció el formato de levantamiento de los índices aédicos de la Secretaría Departamental de Salud de Casanare.

Se revisaron los depósitos artificiales tales como tanques de almacenamiento de agua de las viviendas, llantas, canecas o baldes, recipientes diversos (desechos de alimentos, botellas, tapas, etc.), y depósitos naturales como las axilas de plantas y los huecos en árboles.

Las muestras de larvas y pupas recolectadas en los depósitos, se llevaron al Laboratorio de Entomología Médica Departamental de Casanare, en donde se contaron y se hizo la determinación taxonómica a nivel de especie, usando las claves de Cova, *et al*. ^17^, y Rueda ^18^. Las pupas se mantuvieron vivas hasta que emergieron como adultos para su identificación.

Se calcularon los siguientes indicadores entomológicos: índice larvario de vivienda (IVL), índice larvario de depósito (IDL), índice larvario de Breteau (IBL), índice de pupas de depósito (IDP) e índice de pupas de Breteau (IBP), según la metodología de la Organización Mundial de la Salud (OMS) y del *Special Programme for Research and Training in Tropical Diseases* (*TDR*) ^19^.

### Vigilancia de Aedes albopictus por medio de larvitrampas

Desde el año 2013, se instalaron 12 larvitrampas en tres sitios del área urbana de Yopal: cuatro en la terminal de transporte, cuatro en el aeropuerto y cuatro en la plaza de mercado (figura 1B). Las larvitrampas eran llantas partidas por la mitad, colgadas en árboles a 50 cm del suelo e inundadas con agua de grifo. Las trampas se revisaron cada ocho días y se recolectaban 10 larvas de cuarto estadio por larvitrampa; luego, las trampas se lavaban y se dejaban colocadas hasta la siguiente revisión. Las larvas recolectadas se identificaron taxonómicamente usando las claves anteriormente mencionadas. Los muestreos se hicieron desde enero del 2013 hasta la primera semana de diciembre de 2016.

### Recolección de mosquitos adultos

Los mosquitos adultos se capturaron con el método de atrayente humano protegido: dos investigadores actuaron como voluntarios, expusieron uno de sus brazos y mantuvieron protegido el resto del cuerpo con ropa y con repelente en las zonas expuestas. Las capturas se hicieron entre las 15:30 y las 17:30 horas de los días 13 y 14 de diciembre de 2016, en el peridomicilio de un parqueadero en la zona industrial. Este parqueadero se caracterizaba por la presencia de diferentes depósitos de agua, artificiales y naturales, en donde las llantas desechadas fueron los más frecuentes. El parqueadero tenía, además de la zona de parqueo, un área de viviendas y otra con abundante vegetación que correspondía a la zona de vegetación de bosque de galería ('riparia') de la quebrada o caño Usabar. Se enviaron muestras de los individuos identificados como *A. albopictus* al Instituto Nacional de Salud de Colombia para su confirmación taxonómica.

### Análisis de datos

Se hizo el análisis descriptivo de los datos, utilizando el programa Excel 2013™ de Microsoft. Los indicadores entomológicos para los estadios inmaduros (IVL, IDL y IDP) se presentan como porcentajes con sus respectivos intervalos de confianza del 95 %. Los resultados de las capturas de los mosquitos adultos con atrayente humano se presentan como número de mosquitos por hora.

## Resultados

### Caracterización de criaderos

Se inspeccionaron 955 viviendas y 2.207 depósitos de ocho localidades urbanas y de la zona industrial. Se registraron 150 (15,7 %) viviendas y 219 (9,9 %) depósitos con presencia de larvas del género *Aedes*. Los depósitos más frecuentes en las localidades inspeccionadas fueron los tanques bajos, las canecas y las llantas. Estos también fueron los depósitos con mayor proporción de larvas: tanques bajos, 6,2 %; canecas, 1,4 %, y llantas, 1,4 % (cuadro 1).

**Cuadro 1:** Presencia de larvas de la familia Culicidae en depósitos inspeccionados en ocho localidades de Yopal en diciembre de 2016

Tipo de depósito Depósitos larvas versus				
			*albopictus*	*coronator*	*quinquefasciatus*	total de depósitos *nigripalpus* inspeccionados	
Artificiales Tanque bajo	919	14,8	0,1	0,1	0,3	0	6,2
Diversos	504	3,8	0,4	0	0	0	1,4
Caneca	434	6,9	0	0,2	0,2	0	1,4
Llantas	310	7,7	7,4	0	1,0	0,3	1,4
Natural Árboles y plantas*	40	10,0	0	0	0	0	0,2
Total	2.207						

* Depósitos de agua en huecos del fuste o axilas de plantas.

* Depósitos de agua en huecos del fuste o axilas de plantas.

Se recolectaron 755 larvas de mosquitos. La especie más abundante (n=540) (71,5 %) fue *A. aegypti*, encontrada principalmente en tanques bajos, depósitos naturales y llantas; le siguió *A. albopictus* (n=187) (24,8%), en llantas y diversos. Otras especies poco abundantes fueron *C.quinquefasciatus* (n=24) (3,2 %), *C. coronator* (n=5) (0,7 %) y *C. nig0ripalpus* (n=1) (0,1 %), en tanques bajos, llantas y canecas (cuadro 2).

**Cuadro 2:** Proporción de larvas de especies de la familia Culicidae por tipo de depósitos inspeccionados en las ocho localidades durante diciembre de 2016

					Porcentaje		
Tipo de depósito	Individuos						
		*A. aegypti*		*A.*	*C.*	*C.*	*C.*
				*albopictus*	*coronator*	*quinquefasciatus*	*nigripalpus*
Tanques bajos	411	97,6		0,2	1,0	1,2	0
Llantas	244	24,2		69,7	0	5,7	0,4
Diversos	46	58,7		34,8	0	10,9	0
Canecas	43	97,7		0	2,3	0	0
Árboles y plantas	11	100,0		0	0	0	0

La mayoría (n=170) (69,7 %) de las larvas de *A. albopictus* se recolectaron en llantas, seguidas por recipientes diversos (n=16) (34,8 %), y se encontró una muy baja proporción (n=1) (0,2 %) de larvas en tanques bajos (cuadro 2). La presencia de este vector se registró solamente en dos localidades (barrio Nuevo Hábitat 1 y en la zona industrial) de las ocho inspeccionadas. Además, en las llantas donde se recolectaron las larvas *de A. albopictus* y *A. aegypti*, se encontró una razón entre estas especies de 3:1, respectivamente. Si bien no se determinaron las características fisicoquímicas y biológicas del agua en estos depósitos, se observó hojarasca en descomposición, sedimentos y larvas de la familia Chironomidae.

Por otro lado, los criaderos artificiales infestados con *A. albopictus* estaban ubicados en un tanque bajo del barrio Nuevo Hábitat 1, en la zona urbana de vegetación de bosque de galería ('riparia') de la quebrada Usabar, cerca de la carretera (figura 1B).

Además, se recolectaron 53 pupas en la zona industrial, de las cuales eclosionaron 42 mosquitos de las especies *A. albopictus* (n=32) (76 %) y *A. aegypti* (n=10) (24 %).

### Índices aédicos larvarios y de pupas

Con respecto a los índices aédicos para *A. aegypti*, el mayor IVL registrado fue de 32,1 % en el barrio Siete de Agosto y el menor de 8,1 % en la zona industrial. En cuanto al ID L, el mayor porcentaje se registró nuevamente en Siete de Agosto (22,1 %), seguido de Villa Flor (18,8 %) y la zona industrial (8,0 %). El mayor IBL fue de 54,1 en la zona industrial. El ID P osciló entre 0 % y 18,8 % y el IBP entre 0 y 25, los barrios con mayor IBP fueron Villa Flor (25,0) y Nueva Esperanza (17,1) (cuadro 3).

**Cuadro 3:** Índices aédicos larvarios y de pupas de *Aedes aegypti* en las localidades inspeccionadas en diciembre de 2016

Localidad	Viviendas	DI1	DL2	DP3		Índices larvarios					Índices de pupas				
	inspeccionadas				*IV 4*	*ID 5*			*IB 6*			ID 7	IB 8	
						
					*L*		*L*		*L*			P		P
Siete de Agosto	26	68	15	4	4	22,1(12-32)		53,6	(35-72)	5,9		(0,3-11)	14,3	(1-27)	
Zona industrial	37	323	20	5	5	8,0	(5-9)	54,1	(38-70)	1,5		(0,2-3)	13,5	(3-24)	
Nuevo Hábitat 1	629	1172	121	42	42	10,3	(9-12)	19,2	(16-22)	3,6		(3-5)	6,7	(5-9)	
El Laguito	26	28	3	0	0	10,7	(0-22)	11,5	(0-23)	0			0		
La Bendición	158	396	28	11	11	7,1	(5-10)	17,7	(11-23)	2,8		(2-4)	7,0	(3-11)	
Nueva Esperanza	41	163	20	7	7	12,3	(7-17)	48,8	(33-64)	4,3		(2-7)	17,1	(6-28)	
Llano Lindo	24	41	3	0	0	7,3	(0-15)	12,5	(0-25)	0			0		
Villa Flor	12	16	3	3	3	18,8	(0-37)	25,0	(1-49)	18,8		(0-37)	25,0	(1-49)	

1 Número de depósitos inspeccionados

2 Número de depósitos con larvas

3 Número de depósitos con pupas

4 Índice larvario de vivienda en porcentaje

5 Índice larvario de depósito en porcentajes

6 Índice larvario de Breteau

7 Índice de pupas de depósito en porcentajes

8 Índice de pupas de Breteau

9 Intervalo de confianza del 95 %

Por otra parte, los índices de *A. albopictus* registrados en la zona industrial fueron, IVL en 8,2 %, IDL en 7,7 %, IBL en 67,6, similares a los de *A.aegypti* en esta localidad (cuadro 4).

**Cuadro 4:** Índices aédicos larvarios y de pupas de *Aedes albopictus* en las localidades inspeccionadas en diciembre de 2016

Localidad	Viviendas	DI1	DL2	DP3				Índices larvarios					Índices de pupas		
	inspeccionadas						*IV 4*		*ID 5*		*IB 6*		ID 7	IB 8		
								
							*L*		*L*		*L*		P	P		
Zona industrial	37	323	25	4	8,1		(1-17)9	7,7	(5-11)	67,6	(52-83)	1,2 (0-2)		10,8 (1-21)	
Nuevo Hábitat 1	629	1172	1	0	0,2		(0-0,5)	0,1	(0-0,3)	0,2	(0-0,4)	0		0	
															

1 Número de depósitos inspeccionados

2 Número de depósitos con larvas

3 Número de depósitos con pupas

4 Índice larvario de vivienda en porcentaje

5 Índice larvario de depósito en porcentajes

6 Índice larvario de Breteau

7 Índice de pupas de depósito en porcentajes

8 Índice de pupas de Breteau

9 Intervalo de confianza del 95 %

### Vigilancia de Aedes albopictus con larvitrampas y recolección de mosquitos adultos

Desde enero de 2013 hasta la primera semana de diciembre de 2016, en los tres sitios donde se instalaron las larvitrampas (el aeropuerto, la terminal de transporte y la plaza de mercado), se recolectaron 2.453 larvas, identificadas como *A. aegypti* (n=2.435) (99,3 %), *C. quinquefasciatus* (n=17) (0,69 %) y *C. nigripalpus* (n=1) (0,01 %).

Por otra parte, mediante las capturas con atrayente humano protegido se recolectaron 37 mosquitos, todos clasificados como *A. albopictus*, con un promedio de 7,5 mosquitos/hora y 11,0 mosquitos/hora para cada uno de los dos días de muestreo. Todos los mosquitos fueron recolectados en el área peridomiciliaria ubicada a un metro de distancia de la zona de vegetación de bosque de galería ('riparia') de la quebrada Usabar de la Zona Industrial.

## Discusión

La región de la Orinoquia, conformada por los departamentos de Casanare, Arauca, Meta y Vichada, es una de las regiones colombianas en donde no se había reportado la presencia del vector *A. albopictus*, antes del registro en Yopal ^13^. En los tres últimos departamentos de la región, las Secretarías Departamentales de Salud también han implementado la vigilancia centinela para detectar la presencia de *A. albopictus* desde hace 10 años, aproximadamente (Fuya O. Fortalecimiento de la vigilancia entomológica de *Aedes aegypti* en Colombia para el robustecimiento de la Red Nacional de Entomología, presentación del Instituto Nacional de Salud, 5 de octubre de 2017), logrando identificar otra población de *A. albopictus* en Arauca un año después del primer reporte en Yopal (Armesto Y, Robinson A, Oquendo A, Osto L, Forero L, Cuervo L. Primer registro de *Aedes albopictus* (Skuse) (Diptera: Culicidae) en la frontera colombo-venezolana, departamento de Arauca, Colombia, y sus implicaciones en salud pública. Congreso Colombiano de Entomología, 11-13 de julio de 2018). Por otro lado, no se ha reportado esta especie en Boyacá, departamento vecino del Casanare ubicado en la región andina. Por lo anterior, el presente reporte es el primer registro de *A. albopictus* en Yopal, Casanare, y en la región de la Orinoquia colombiana.

Es pertinente mencionar que en Venezuela, país limítrofe con esta región colombiana, tampoco se ha reportado la especie en los estados cercanos, como Amazonas o Apure ^20^^-^^22^. El reporte más cercano a Colombia, se hizo en el municipio de Sucre del estado Bolívar, ubicado en el centro de este país. Asimismo, otros reportes de *A. albopictus* en Venezuela se dieron en el centro-norte y noroccidente (estados de Aragua, Monagas, Guárico, Carabobo, Trujillo y el Distrito Capital) entre los años 2009 y 2017 ^20^^-^^22^.

En cuanto al departamento de Casanare, este es considerado como zona endémica para el dengue, con transmisión activa de Zika y chikungunya, virus transmitidos principalmente por *A. aegypti* en el área urbana. Teniendo en cuenta la ecoepidemiología de *A. albopictus*, su introducción se considera como un nuevo factor de riesgo para la transmisión de estas arbovirosis en el departamento y en la región. Esta especie tiene la capacidad de transmitir cepas del virus del chikungunya, como sucedió en el brote en la provincia de Ravenna, Italia, y en la epidemia de la isla Reunión en el océano Índico, a causa de la cepa E1-A226V ^2^^,^^23^. También, transmite los cuatro serotipos del virus del dengue, como ocurrió en las epidemias de dengue en la isla Seychelles y en Guandong, China ^3^. 

Cabe señalar que *A. albopictus* puede intervenir como vector en la urbanización de los virus Mayaro y de la fiebre amarilla ^6^, y transmitir el virus de la encefalitis equina del este ^24^, alfavirosis que tuvo un comportamiento activo en el 2016 con 57 casos en equinos en Casanare, situación que forzó al departamento a declarar diferentes zonas en cuarentena ^25^.

Con respecto a los índices aédicos, en general, el IVL fue superior a 5 % en las localidades inspeccionadas, y el IBL osciló entre 0,2 y 67,6, valores que algunos autores consideran altos. Por ejemplo, la OPS considera un IVL superior a 5 % como un valor de alto riesgo para la transmisión de dengue ^26^. Sin embargo, este valor puede cambiar debido a las variaciones ambientales entre las zonas. En el caso de las zonas tropicales, se había establecido un IV L del 10 % como un límite seguro (pocas probabilidades de transmisión de dengue); posteriormente, este valor se cambió a 5 % ^27^^,^^28^.

A pesar de lo anterior, la transmisión de dengue fue reportada por debajo del 3 % en Salvador y en el Brasil, y por debajo del 1 % en Singapur ^29^.

Asimismo, para los valores del índice de Breteau entre 35 y 50 se consideraban como de alto riesgo en las zonas de transmisión ^27^.

Posteriormente, en La Habana (Cuba), el límite seguro del índice de Breteau fue establecido en 4 ^30^. No obstante, Bowman, *et al*. ^29^, encontraron reportes de transmisión de dengue con un índice de Breteau por debajo de 5, en su revisión.

Sin embargo, esta correlación entre los indicadores entomológicos y la transmisión del dengue todavía no es clara. El tema fue recientemente debatido por Cromwell, *et al*. ^31^, debido a que la utilidad de los indicadores aédicos es limitada, porque no valora otras variables importantes que existen en la relación entre el humano y el vector, como las características de la vivienda, el estrato social y el desplazamiento humano.

Por otro lado, en los criaderos inspeccionados en la zona industrial, en su mayoría artificiales, predominaban las larvas de *A. albopictus* y de la familia Chironomidae, familia con la cual no hay competencia por los recursos ^7^.

Durante las capturas con atrayente humano protegido, *A. albopictus* fue el único vector recolectado, en un muestreo limitado a dos días y con una duración de dos horas diarias. A pesar de lo anterior, la cantidad de mosquitos capturados en este estudio fue mayor a lo registrado en Leticia (Amazonas) y en Venezuela. En Leticia se recolectaron ocho mosquitos durante dos días en horario de 8:00 am a 5:30 pm ^9^ y, en el municipio Sucre del estado Bolívar en Venezuela, se recolectaron cinco mosquitos con atrayente humano y con el uso de la trampa Mosquito Magnet™ durante siete horas de muestreo en dos días ^22^.

En Colombia no se han registrado resultados de capturas con la metodología de atrayente humano protegido, con los que se pueda establecer una comparación, a excepción de los registrados para *A. aegypti* en Guaduas (Cundinamarca). En este estudio, se encontró que la mayor actividad de picadura de *A. aegypti* es de 10:00 a.m. a 11:00 a.m. y de 4:00 p.m. a 6:00 p.m.; se recolectaron entre 38 y 40 mosquitos en 10 viviendas durante la temporada seca ^32^.

Es de agregar que, en el lugar de muestreo, solamente se capturaron adultos de *A. albopictus*, a pesar de que cerca de este sitio existían depósitos con larvas de *A. aegypti*. . Esto es un indicio del hábito antropofágico de *A. albopictus* en el área de estudio. Aunque el alcance de este estudio no permitía determinar el comportamiento de alimentación del vector, en otros estudios se ha confirmado el alto nivel de antropofagia de *A. albopictus*. Kamgang, *et al*. ^33^, en Camerún, reportaron que 92 % de los mosquitos analizados habían ingerido solamente sangre humana; mientras que Delatte, *et al*. ^34^, reportaron que 89 % de los mosquitos capturados en la isla Reunión habían ingerido esta sangre. En ambos estudios, entre el 81 % y el 100 % de los mosquitos de *A. albopictus* se capturaron con atrayente humano en el peridomicilio. Esto evidencia un comportamiento exofágico de *A. albopictus* en esta región, a diferencia de *A. aegypti*^34^^,^^35^.

En los depósitos principalmente periurbanos con presencia de *A. albopictus*, se encontró un aparente desplazamiento de *A. aegypti* por esta especie, evidenciado por la razón 3 a 1 de *A. albopictus* con relación a *A. aegypti*. Lo anterior coincide con los resultados de Barrera ^36^ en experimentos en el laboratorio y observaciones de campo en Brasil, en donde se encontró que las poblaciones de *A. aegypti* decrecieron después de 22 años de la introducción de *A. albopictus*. En Estados Unidos, también se ha reportado que *A. albopictus* ha competido y desplazado a *Ochlerotatus triseriatus*, especie nativa de dicho país ^1^.

Se cree que el desplazamiento de *A. aegypti* se debe a un fenómeno denominado ‘interferencia reproductiva’. Este fenómeno consiste en que los machos de *A. albopictus* pueden copular con hembras de *A. aegypti* y, durante su apareamiento, estos inyectan sustancias de la glándula accesoria que le impiden a la hembra posteriores apareamientos y disminuyen su actividad locomotora diurna. Lo anterior no ocurre entre machos de *A. aegypti* y hembras de *A. albopictus*^37^^-^^39^, en consecuencia, el éxito reproductivo de *A. aegypti* disminuye.

Según Rey y Lounibos ^40^, el desplazamiento competitivo también se puede atribuir a la competencia de las dos especies por el mismo nicho ecológico, que reduce la población de una de las especies, principalmente en los criaderos periurbanos, por la disponibilidad de agua, por características ambientales como la precipitación y la humedad, o por ambos factores.

Por consiguiente, es recomendable hacer un seguimiento temporal de la densidad y de los índices entomológicos de las poblaciones de *A. albopictus* y de *A. aegypti*, en diferentes épocas del año, para verificar la influencia de *A. albopictus* sobre *A. aegypti.* También, se debe establecer si hay correlaciones positivas entre las variables climáticas y la abundancia del vector, pues existe una importante correlación entre el aumento de las precipitaciones y la abundancia de *A. albopictus*^41^. De igual forma, se debe vigilar la transmisión de arbovirosis durante las épocas cuando las poblaciones del vector son favorecidas por las condiciones ambientales.

El sitio donde se encontró *A. albopictus* se caracteriza por el gran flujo de vehículos de transporte de carga, originarios de diferentes lugares del país, y por la acumulación de llantas desechadas por los propietarios de estos vehículos. Este pudo haber sido el medio de introducción del vector a la región, como ocurrió en los Estados Unidos (Lounibos LP. Ecoepidemiologia del dengue: relevancia de dos vectores invasores. Biomédica. 2011;31(Supl. 3):50-9). Por lo anterior, es difícil establecer el tiempo exacto en el cual *A. albopictus* llegó al municipio de Yopal.

Actualmente, se desarrollan actividades de vigilancia entomológica para *A. aegypti* en el departamento de Casanare, pero se recomienda incluir un nuevo criterio para la vigilancia, con el fin de detectar a las poblaciones de *A. albopictus* en las zonas periurbanas. Principalmente, se deben inspeccionar los depósitos artificiales y naturales.

Cabe señalar que las larvitrampas no fueron un método eficaz para detectar *A. albopictus* en Yopal. Ciertamente, tampoco ha sido el método para los primeros registros del vector en poblaciones como Leticia, Buenaventura, La Tebaida, Medellín, Istminia e, incluso, Cali, donde solo se detectó después de cuatro años de instaladas y del ingreso previo a municipios cercanos, como Buenaventura y Dagua ^9^^-^^14^.

Sin embargo, no se deben descartar las larvitrampas como método de apoyo, pero modificando algunas de sus características, particularmente, reemplazando el agua del grifo por agua con alto contenido de materia orgánica. Lo anterior se afirma con base en las características fisicoquímicas del agua contenida en depósitos similares a las llantas y en las llantas usadas como larvitrampas en el presente estudio, en donde los resultados de la detección de *A. albopictus* fueron opuestos.

En las larvitrampas en donde no se detectó *A. albopictus*, se usó agua de grifo sin sedimentos y concentraciones aparentemente bajas de compuestos nitrogenados. Por el contrario, en los depósitos en llantas, en donde se reportó el mayor número de larvas de *A. albopictus*, el agua presentó características fisicoquímicas similares a la de los criaderos naturales: agua de lluvia, con hojarasca en descomposición, sedimentos y larvas de la familia Chironomidae.

Este tipo de depósito tiene altos contenidos de materia orgánica o nitrógeno orgánico y plancton, condiciones preferidas por *A. albopictus* debido a su origen selvático ^3^^,^^7^. Además, se deben usar ovitrampas y trampas adhesivas, como trampas de emergencia para mosquitos ^42^, para complementar la vigilancia de esta especie y detectar su introducción a nuevos sitios.

A lo anterior se añade, a nivel local, priorizar la zona de vegetación de bosque de galería ('riparia') de la quebrada Usabar. Este hábitat favorece la expansión del vector en el municipio y su desplazamiento sobre la margen del cuerpo de agua, convirtiéndose en un factor de riesgo para las personas. De acuerdo con el plan de ordenamiento territorial de Yopal ^43^, la margen de la quebrada en mención colinda con el área programada para la expansión del suelo urbano en donde se están estableciendo nuevos asentamientos humanos.

Finalmente, para los departamentos de la Orinoquia colombiana y, específicamente para Casanare, es recomendable intensificar la vigilancia entomológica para detectar nuevas poblaciones de *A. albopictus*, incluyendo los sitios de afluencia de los vehículos provenientes de diferentes partes del país y donde se hace su mantenimiento. La razón es que, como se demostró en el presente estudio, estos sitios favorecen el establecimiento de poblaciones de *A. albopictus,* pues pueden mantener un gran número de criaderos potenciales. Además, existe la probabilidad de la dispersión del vector mediante las llantas transportadas por los vehículos y no solo por la proximidad geográfica con otros departamentos donde circula el vector.
